# Olive Oil Waste as a Source of Functional Food Ingredients: Assessing Polyphenolic Content and Antioxidant Activity in Olive Leaves

**DOI:** 10.3390/foods13020189

**Published:** 2024-01-06

**Authors:** Carolina L. Ronca, Sara S. Marques, Alberto Ritieni, Rafael Giménez-Martínez, Luisa Barreiros, Marcela A. Segundo

**Affiliations:** 1 LAQV, REQUIMTE, Department of Chemical Sciences, Faculty of Pharmacy, University of Porto, 4099-002 Porto, Portugal; carolinaronca@correo.ugr.es (C.L.R.); scmarques@ff.up.pt (S.S.M.); lbarreiros@ff.up.pt (L.B.); 2 Department of Pharmacy, Faculty of Pharmacy, University of Naples “Federico II”, 80138 Naples, Italy; 3 Department of Nutrition and Bromatology, School of Pharmacy, University of Granada, 18012 Granada, Spain; rafaelg@ugr.es; 4 School of Health, Polytechnic Institute of Porto, 4200-072 Porto, Portugal

**Keywords:** circular economy, olive oil waste valorization, sustainable extraction, functional food ingredients, Mediterranean diet, bioactive compounds, antioxidant capacity

## Abstract

Around two million tons of olive oil are produced in Europe annually, with Portugal being among the top five European olive oil-producing countries. Olive oil production results in a substantial amount of waste in the form of olive leaves. These discarded olive leaves contain valuable phenolic compounds with antioxidant, anti-inflammatory, hypoglycaemic, neuroprotective, and antiproliferative properties. Due to their richness in polyphenols with health-promoting properties, olive leaves can be considered a potential functional food ingredient. Thus, sustainable practices for reusing olive leaf waste are in demand. In this study, the polyphenolic content in olive leaves from different Portuguese locations was determined using HPLC-UV-Vis after defining the best fit-for-purpose liquid extraction strategy. The differences in the in vitro antioxidant activity in these samples were determined by several methodologies based on radical scavenging (against 2,2′-azino-bis-3-ethylbenzthiazoline-6-sulphonic acid (ABTS), 2,2-diphenyl-2-picrylhydrazyl (DPPH), and peroxyl radical (ORAC)) and on reducing properties (cupric-reducing antioxidant capacity (CUPRAC), and Folin–Ciocalteu assay (FC)), to unveil the relationship between the profile and quantity of polyphenols with antioxidant mechanisms and their capacity. At last, the stability of extracted compounds upon lyophilization and exposition to surrogate biological fluids was assessed, envisioning the future incorporation of olive leaves extracted compounds in food products.

## 1. Introduction

Olive oil is the keystone of the Mediterranean diet. This vegetable oil, which represents a healthy fat source, is largely consumed worldwide, partially due to its known associated health benefits, such as antioxidant [1,2], anti-inflammatory [2], hypoglycaemic [3], and neuroprotective effects [4,5,6]. Every year, approximately 3 million tons of olive oil are produced worldwide [7,8]. From these, ≈2 million tons were produced in European countries, mainly in Spain (66%), Italy (15%), Greece (13%) and Portugal (5%) in 2020 [9]. Also, European countries rank among the top olive oil consumers, with consumption of around 1.5 million tons of olive oil per year.

Olive oil derives from the fruit of *Olea europaea* L., whose main agriculture area (≈98%) is located in Mediterranean countries [8,10]. Portugal generated 784,340 tons of olives in 2021 [11], 97% of which was dedicated to olive oil production, which is currently in the top 5 of European producers and in the top 10 of the world’s largest producers [12,13]. The wide geographical areas of olive plantations in Portugal are located in Alentejo (50%), Trás-os-Montes (23%), Beira Interior (14%), and Ribatejo and Oeste (7.5%), with some activity in the field also present in Algarve and Beira Litoral regions [11]. Due to the extensive development in this sector, along with the suitability of the ground and the remarkable Portuguese climatic conditions, Portugal is an emerging country in the olive oil production area [14]. In fact, it is estimated that by 2030, Portugal will be the third-largest olive oil producer in the world [12]. The expansion in olive oil production and, consequently, on olive tree harvesting, however, poses environmental challenges, as this activity generates high amounts of waste (such as olive leaves and wood) and by-products (olive pomace, mill wastewater, and olive stones) [15]. Olive leaves are a significant waste product generated during the olive oil production process. During pruning and harvesting processes, approximately 25 kg/year of twigs and leaves are produced per tree, which represents 10% of the total weight of processed olives [8,10]. Most of the waste from olive leaves is discarded, posing an environmental issue. Considering that olive leaves are a valuable source of phenolic compounds [8] and are aligned with the principles of circular economy in minimizing environmental impact and maximizing the use of resources, the potential of olive leaf waste has been exploited [8,16]. Indeed, olive leaves are a rich source of polyphenolic compounds such as secoiridoids (e.g., oleuropein, hydroxytyrosol, verbascoside), flavonoids (e.g., rutin, luteolin, quercetin, apigenin, diosmetin), and isoflavonoids, with demonstrated therapeutic benefits [17,18] both in vitro and in vivo [18,19,20,21].

Rutin and luteolin have shown hypoglycaemic and antioxidant activities, while quercetin has demonstrated anti-inflammatory and anti-tumoral effects [22]. Likewise, oleuropein, a secoiridoid compound constituting the main polyphenol in olive leaves, has shown protective effects against oxidative stress [23], anti-inflammatory activity [24], and positive effects on the control of body weight [25], glycemia [26], and intestinal microbiota [27,28]. In recent years, increased attention has also been given to hydroxytyrosol, which is the main metabolite of oleuropein. This compound has shown beneficial effects in protecting cellular lipids, proteins, and DNA from oxidative damage and preventing effects from degenerative, cardiovascular, or carcinogenic diseases [29]. Apart from these, verbascoside has demonstrated important antioxidant properties [30].

Considering the valuable profile of olive leaves as a source of compounds with therapeutic properties, sustainable practices to promote the reuse of olive leaf waste from olive oil production towards the extraction of these compounds are in demand.

Several methods have been applied to extract polyphenolic compounds from plants using different extracting solvents [31] (e.g., water, organic solvents, or hydroalcoholic mixtures) such as liquid–liquid extraction [32], microwave-assisted extraction [21,31,33], pressurized liquid extraction [34], supercritical fluid extraction [35], solid-phase extraction [31], and ultrasound-assisted extraction [36]. Currently, there is a concern about accomplishing greener procedures when extracting compounds of interest from agro-food matrices such as olives leaves, by employing greener extraction solvents such as water, 70:30 (*v*/*v*) water/ethanol solutions or deep eutectic solvents [37]. For olive leaves, in particular, recent works have proposed pressurized propane [38] or isopropanol water [39] for the extraction of bioactive compounds. The first approach is not adequate for polyphenols as they do not have a low boiling point. The second one is not compatible with further direct utilization for human or animal consumption, as also highlighted for other commonly employed solvents, such as methanol, ethyl acetate, hexane, diethyl ether, chloroform, and butanol [40].

In this work, the recovery of polyphenols from olive leaves was intended using solid–liquid extraction based on a solvent that contributes to the greenness and sustainability of the process, along with compatibility for food applications. The best extraction solvent was assessed by monitoring the polyphenols’ profile through high-performance liquid chromatography (HPLC) coupled with UV-Vis detection. Then, the antioxidant capacity for the different samples of olive leaves was assessed using several in vitro methodologies, namely Folin–Ciocalteu, the cupric-reducing antioxidant capacity (CUPRAC), 2,2-diphenyl-1-picrylhydrazyl radical scavenging capacity (DPPH), 2,2′-azinobis-(3-ethylbenzothiazoline-6-sulfonic acid) (ABTS) and oxygen radical absorption capacity (ORAC). The relationship between quantitative analysis via HPLC and the antioxidant capacity assessed by diverse methodologies was assessed to elucidate additive and synergistic effects. Also, bioaccessibility studies using the richest extract were performed to evaluate the feasibility of extracted compounds for future incorporation into food products upon lyophilization processes.

## 2. Materials and Methods

### 2.1. Chemicals and Solutions

3-hydroxytyrosol (purity 98%), (+)-catechin hydrate, oleuropein, (+)-pinoresinol, caffeic acid (purity ≥ 98%), gallic acid (purity ≥ 98%), rutin hydrate (purity ≥ 94%), quercetin (purity ≥ 95%), and verbascoside (purity ≥ 99%), were obtained from Sigma-Aldrich (Sigma-Aldrich, St. Louis, MO, USA). Luteolin (purity ≥ 97%) was acquired from Alfa Aesar GmbH & Co (Alfa Aesar, Karlsruhe, Germany). Ultrapure water (resistivity > 18 MΩ cm) was obtained from the AriumPro system (Sartorius, Göttingen, Germany) and used in the preparation of all aqueous solutions. Methanol (MeOH, LiChrosolv HPLC grade) and acetonitrile (ACN, LiChrosolv HPLC grade) were purchased from VWR Chemicals (Radnor, PA, USA) and used in the preparation of HPLC and extraction solutions. Formic acid (purity ≥ 95%) and dimethyl sulfoxide (DMSO, purity ≥ 99.5%) were obtained from Sigma-Aldrich (Sigma-Aldrich, St. Louis, MO, USA). Ethanol absolute (EtOH) was obtained from Chem-Lab NV (Zedelgem, Belgium). Stock solutions of 3-hydroxytyrosol (1 mg mL^−1^), catechin (1 mg mL^−1^), oleuropein (1 mg mL^−1^), and gallic acid (1 mg mL^−1^) were prepared in ultrapure water. Stock solutions of rutin hydrate (1 mg mL^−1^), caffeic acid (1 mg mL^−1^), pinoresinol (0.3 mg mL^−1^), and luteolin (1 mg mL^−1^) were prepared in MeOH:water (70:30, *v*/*v*). A quercetin stock solution (1 mg mL^−1^) was prepared in DMSO:water (50:50, *v*/*v*). All stock solutions were stored at 4 °C and protected from light. From this, daily working standard solutions (0.25–25 mg L^−1^) were prepared in 85:7.5:7.5 (*v*/*v*/*v*) and a 0.02% (*v*/*v*) formic acid aqueous solution:MeOH:ACN. For comparison purposes, calibration curves at 80% (*v*/*v*) methanol with 0.02% (*v*/*v*) formic acid, and 80%, 50%, and 10% (*v*/*v*) ethanol with 0.02% (*v*/*v*) formic acid were also performed.

Regarding the antioxidant studies, Folin & Ciocalteu’s phenol reagent, 2,2-Diphenyl-1-picrylhydrazyl (DPPH), 2,2′-azino-bis(3-ethylbenzothiazoline-6-sulfonic acid) diammonium salt (ABTS), potassium persulfate, neocuproine (Nc) hydrochloride monohydrate, copper(II) chloride dihydrate, fluorescein sodium salt, 2,2′-azobis(2-amidinopropane) dihydrochloride (AAPH), (±)-6-hydroxy-2,5,7,8-tetramethylchromane-2-carboxylic acid (Trolox), ammonium acetate and potassium phosphate were acquired from Sigma. Sodium acetate trihydrate and sodium carbonate decahydrate were purchased from Fluka (Buchs, Switzerland).

For the bioaccessibility studies, gastric and intestinal simulated fluids were prepared according to the US Pharmacopeia 38—National Formulary 33 [41]. Gastric-simulated fluid (50 mL) was prepared by dissolving 0.1 g of NaCl (VWR Chemicals, Radnor, PA, USA), 0.16 g of porcine pepsin (Sigma-Aldrich, St. Louis, MO, USA) and 0.68 mg of lecithin (Sigma-Aldrich) in water up to 40 mL, followed by pH adjustment to 1.2 with HCl 2 M (VWR Chemicals) and volume completion. The intestinal simulated fluid was prepared from the gastric digest by adding this to a phosphate-buffered solution (0.2 M, pH 6.8), which was prepared by dissolving 0.85 g of potassium phosphate dibasic (Sigma-Aldrich) and 0.69 g of potassium phosphate monobasic (Sigma-Aldrich) in 100 mL of ultrapure water and pancreatin (Sigma-Aldrich).

### 2.2. Olive Leaves Samples

Fresh olive leaves from different locations in Portugal (Table S1) were collected. The leaves were dried at room temperature and protected from light until a constant weight was obtained, detailed as follows: 7 g of each sample was segregated from the pool of leaves and weighed upon arrival in the lab. During the drying procedure, each aliquot was frequently weighed (e.g., once a day) until no further mass variation was observed for three days. The initial content of olive leaves moisture was determined considering the mass decrease upon drying. Therefore, the initial moisture content ranged from 12 to 34%.

### 2.3. Preparation of Olive Leaves Extracts

Twenty grams of dried olive leaves from each sample were collected and powdered, resorting to a conventional coffee mill (Model Nevir, 230 V, 130 W, Alfonso Gomez, Madrid, Spain). From the resulting powder, 0.5 g was weighted. To this, 12.5 mL of a 50:50 (*v*/*v*) EtOH:water with 0.1% (*v*/*v*) formic acid solution (extraction solvent C) was added and mixed via vortex for one minute. Then, this preparation was placed in an ultrasound bath for 15 min, followed by orbital agitation at 210 rpm, 20 °C for 20 min (incubator shaker RO 10 S000, IKA^®^, Staufen im Breisgau, Germany). The resultant solution was centrifuged at 4750× *g* at 20 °C for 20 min (ALLEGRA X-15R Centrifuge, Beckman Coulter, Landshut, Germany), followed by supernatant collection in a volumetric flask. The resultant pellet from this procedure was submitted to a second extraction sequence, and the corresponding supernatant was collected and added to the first portion. A final volume of 25 mL was ensured by volume completion in a volumetric flask of 25 mL. The same procedure was applied using 80:20 (*v*/*v*) MeOH:water with 0.1% (*v*/*v*) formic acid; 80:20 (*v*/*v*) EtOH:water with 0.1% (*v*/*v*) formic acid; 10:90 (*v*/*v*) EtOH:water with 0.1% (*v*/*v*) formic acid; and only water with 0.1% (*v*/*v*) formic acid solutions as the extraction solvents. For simplicity, these solutions were named extraction solvents A, B, D, and E, respectively. The experiment using 80:20 (*v*/*v*) MeOH:water with 0.1% (*v*/*v*) formic acid was included only for comparison purposes, as MeOH is not adequate concerning extraction greenness.

Each olive leaf extract was submitted to centrifugation at 18,000× *g* for 10 min at 4 °C (Microfuge 22R Centrifuge, Beckman Coulter, Landshut, Germany) before analysis using HPLC-UV-Vis.

### 2.4. Chromatographic Conditions

Chromatographic analysis was performed on the Jasco HPLC system (Easton, PA, USA) equipped with a PU-2089 pump, AS-2057 autosampler, LC-Net II/ADC controller, and Jasco MD-2015 photodiode array detector. Standards and samples were injected (20 μL) into a reversed-phase Kinetex^®^ core–shell C18 column (250 × 4.6 mm; 5 μm particle size; 100 A). 

Separation was performed in a gradient mode by mixing in-line solutions (A) 0.02% (*v*/*v*) formic acid (pH ≈ 3.0) aqueous solutions and (B) 50:50 (*v*/*v*) MeOH:ACN with 0.02% (*v*/*v*) formic acid at a 0.8 mL min^−1^ flow rate. Elution started with 15% of B (0–4 min), followed by an increase from 15 to 60% of B (4–26 min). Then, a higher organic content (40:60 (*v*/*v*) A:B) was maintained for 5 min, followed by a return to initial conditions (31–36 min) and 10 min of column equilibration (36–46 min) before the next run. Determinations were performed at room temperature. UV detection was performed with spectra acquisition from 200 to 400 nm, with the quantification of phenolic compounds at 280, 320, and 350 nm (Figure S1). The quantification of olive leaf-extracted polyphenols was performed via the interpolation of peak area values for each compound in the calibration curve obtained with standards (Table S2). Analyzed extracts were fortified with a standard mixture at 5 mg L^−1^ for the assessment of compound identification, complemented by UV spectra inspection.

Olive leaf extracts were analyzed directly using HPLC after centrifugation at 18,000× *g* for 10 min at 4 °C (Microfuge 22R Centrifuge, Beckman Coulter, Germany). Also, olive leaf extracts were analyzed after lyophilization (Benchtop Freeze Dryer, Telstar, Spain; conditions: 68 h of lyophilization at 0.02 mBar and −80 °C), and the results were compared to those obtained prior to the lyophilization procedure.

### 2.5. Antioxidant Assays

Antioxidant capacity assays were performed in a 96-well microplate reader (Synergy HT or Cytation3^®^ microplate reader, both from Bio-Tek Instruments, Winoosky, VT, USA), controlled by Gen5 (Bio-Tek Instruments) software (version 2.01.14 (Synergy HT) or version 2.06 (Cytation3^®^)). Assays were performed in triplicate or quadruplicate. Different extract dilutions (a minimum of 3) were tested by reducing and scavenging antioxidant capacity assays, as detailed below. Moreover, the antioxidant capacity for standards from the phenolic compounds under study was also evaluated for each methodology.

#### 2.5.1. CUPRAC: Cupric-Reducing Antioxidant Capacity Assay

The CUPRAC assay was performed as described elsewhere [42] with slight modifications. Briefly, 50 μL of an aqueous copper (II) solution (10 mM), 50 μL of an aqueous neocuproine (Nc) solution (7.5 mM), and 50 μL of ammonium acetate (1.0 M, pH 7.0) were added to the microplate wells to form the CUPRAC (Cu(II)-Nc) chromogenic reagent. Then, 100 μL of olive leaf extracts at different concentration levels (corresponding to 10–1000× dilution) or Trolox standard solutions (15–200 μM), both prepared in a 10% (*v*/*v*) ethanol aqueous solution, were added to the wells (4 replicates for each dilution). The antioxidant capacity, based on the reduction in Cu(II)-Nc to Cu(I)-Nc with the formation of a yellow-orange product, was based on the measurement of absorbance values at 450 nm during 60 min. Blank control experiments were also performed via the addition of 100 μL of an ethanolic solution (10%, *v*/*v*) instead of the sample or Trolox. The antioxidant capacity values were expressed in Trolox equivalents, as mmol Trolox g^−1^ of the sample, by interpolating the absorbance values obtained for the samples in the Trolox standard curve.

#### 2.5.2. Folin–Ciocalteu-Reducing Assay

The Folin–Ciocalteu (henceforward referred to as Folin) method, used to measure total phenolic content [42], was performed according to [42,43], with some changes: briefly, 150 μL of sample aqueous solutions at different concentration levels (10–1000× dilution) or the gallic acid standard aqueous solution (6–90 μM) were placed in each well. Thus, 50 μL of the Folin reagent was added, followed by 100 μL of the carbonate solution (9% *w*/*v*, pH ≈ 10). A reagent blank was performed, replacing the sample with 150 μL of water. The capacity of phenolic compounds to reduce the phosphomolybdic/phosphotungstic acid reagent was monitored at 760 nm (room temperature) every 10 min for 2 h. The results were expressed as mmol of gallic acid g^−1^ in the sample by interpolating the absorbance values measured for samples in the gallic acid calibration curve.

#### 2.5.3. DPPH: 2,2-Diphenyl-1-picrylhydrazyl Radical Scavenging Capacity Assay

The microplate DPPH antioxidant assay was performed according to [42]. Briefly, 150 μL of olive leaf extracts at different dilutions (10–1000× dilution) or Trolox standard solutions (9–70 μM) prepared in 50% (*v*/*v*) ethanol/water was added to the microplate wells. To these, 150 μL of the DPPH^•^ solution (prepared at 50% *v*/*v* ethanol) at a concentration of 30–210 μM was added to render an initial absorbance value of ≈0.90, as described elsewhere [42]). The antioxidant capacity was monitored by measuring the decrease in the purple chromogenic substrate 2,2-diphenyl-1-picrylhydrazyl (DPPH^•^) every 10 min, with absorption at 515 nm, to the corresponding pale yellow hydrazine, during a 120 min reaction time [42,44,45]. Blank experiments were conducted in which 150 μL of the ethanolic solution (50%, *v*/*v*) was placed instead of a sample, or Trolox was performed to monitor the stability of the DPPH^•^ radical during the reaction time. Net absorbance values were calculated for samples and for Trolox as the difference in the absorbance values obtained in their presence in relation to those found in blank experiments. Olive leaf sample extracts’ antioxidant capacity was expressed as mmol Trolox g^−1^ of the sample by interpolating the values obtained for the samples in the Trolox standard curve.

#### 2.5.4. ABTS: 2,2′-Azinobis-(3-ethylbenzothiazoline-6-sulphonate) Radical Cation Scavenging Capacity Assay

The antioxidant capacity to scavenge ABTS^•+^ radical (ABTS assay) was performed according to [42,43] with slight modifications. The ABTS^•+^ radical solution was prepared 12–16 h prior to use by mixing equal volumes of the ABTS aqueous solution (7 mM) and potassium persulfate aqueous solution (2.45 mM) [42]. Before being employed in the assay, this solution was diluted in an acetate buffer (50 mM, pH 4.6) to yield 88–350 mM solutions and to perform a calibration curve prior to the assay. From this calibration curve, the concentration of an ABTS^•+^ radical solution prepared in acetate buffer to use in the assay (concentration yielding an initial absorbance value of 0.90 ± 0.02) was defined.

Then, in the microplate wells, 150 μL of olive extract dilutions (10–1000× dilution) or Trolox standard solutions (7–50 μM) prepared in a 10% (*v*/*v*) ethanolic solution were placed, followed by the addition of 150 μL of the ABTS^•+^ radical solution prepared in acetate buffer (50 mM, pH 4.6). 

Antioxidant capacity was assessed via the reduction in the green-blue-colored ABTS^•+^ radical to a colorless product through absorbance monitoring at 734 nm every 10 min for 5 h. The absorbance of the ABTS^•+^ radical (blank experiment) alongside the reaction time was monitored by adding 150 μL of a 10% (*v*/*v*) ethanolic solution instead of a sample or Trolox. Net absorbance values were determined for Trolox and olive leaf extracts by subtracting the absorbance values after 5 h from those found in the blank experiments. The antioxidant capacity values of olive leaf extracts were expressed as mmol Trolox g^−1^ of the sample.

#### 2.5.5. ORAC: Oxygen Radical Absorbance Capacity Assay

The ORAC assay was performed as described elsewhere [42,46] with some modifications. Primarily, 20 μL of olive leaf extracts at different dilutions (10–1000×) or Trolox standard solutions (10–200 μM) were incubated with a fluorescein solution (117 nM) prepared in a phosphate buffer (75 mM, pH 7.4), for 15 min, at 37 °C. Then, 60 μL of the AAPH solution (40 mM, prepared in phosphate buffer, pH 7.4) was added to the microplate wells. Blank experiments were performed by assessing fluorescein oxidation in the absence of a sample/Trolox. Likewise, control experiments to monitor fluorescein’s stability alongside the reaction time were performed by replacing the sample/Trolox and AAPH with phosphate buffer. Fluorescence values (λ_exc_ 485 nm, λ_em_ 528 nm) were measured every minute for 4 h (240 min) at 37 °C. The area under the curves (fluorescence vs. time) was calculated for Trolox standard solutions and for the olive leaf extracts. Antioxidant capacity values were expressed in Trolox equivalents, as the mmol Trolox g^−1^ of the sample, by interpolating the area under the curve obtained for the sample extracts in the Trolox standard curve.

#### 2.5.6. Samples Theoretical Trolox Equivalent Antioxidant Capacity (TEAC) Values

The relative antioxidant capacity of each phenolic compound under assessment in this study in relation to Trolox (TEAC) was determined according to [47] for the CUPRAC, ABTS, DPPH, and ORAC antioxidant capacity assays. For the Folin assay, these were determined in relation to gallic acid. These values were calculated by dividing the slope of the linear responses (calibration curve—absorbance signal vs. concentration) obtained for the standard compounds by that obtained for the Trolox calibration curve [47]. Then, the experimental TEAC values found for each compound were multiplied by the concentration of the compound on the olive leaf extracts found using HPLC, and the theoretical TEAC value for each sample was obtained by summing all individual contributions [47].

### 2.6. Bioaccessibility Assays

Thirty-five milligrams of the olive leaf extract powder (obtained via lyophilization) was incubated with 5.3 mL of gastric fluid for 2 h (pH = 2, containing pepsin) at 37 °C under orbital mixing (incubator shaker RO 10 S000, IKA^®^, Staufen im Breisgau, Germany). After incubation with the surrogate gastric fluid, the powder was submitted to the intestinal fluid surrogate by adding this to the gastric digest with 2.7 mL of the phosphate buffer (pH 6.8, 0.2 M), followed by pH adjustment to 6.8 using a NaOH (Pronalab, Lisboa, Portugal) solution at 2 M, and the addition of 80 mg of pancreatin to the previous solution. Intestinal digestion was performed for 4 h at 37 °C under orbital mixing. Prior to HPLC analysis, the result from olive leaf extract incubation was submitted to a sample treatment procedure [48]. Briefly, ACN was added to samples until an organic solvent content of 80% was obtained (*v*/*v*). After 30 s of vortexing, 1.44 g of zinc sulfate was added. The resultant was then vortexed and centrifuged (18,000× *g*, 10 min, 4 °C). The supernatant was collected and diluted to match the mobile phase composition before HPLC analysis.

Experiments in which olive leaf extract powder samples were submitted only to gastric surrogate fluid were also performed. Moreover, control experiments (with no olive leaf extract) in which gastric and intestinal simulated fluids were fortified with the polyphenols under study (gallic acid, hydroxytyrosol, catechin, caffeic acid, verbascoside, rutin, oleuropein, pinoresinol, quercetin, luteolin) at the concentration levels found in the extracts under analysis were also performed. A similar experiment to the latter was also executed, replacing the fluids with water to evaluate the stability of the compounds to the 37 °C used during the assays.

### 2.7. Statistical Analysis

Values are presented as the mean ± standard deviation (S.D.) for each assay. Each extract was analyzed in triplicate (*n* = 3) using HPLC. For the antioxidant assays, each extract dilution or standard compound was analyzed in quadruplicate (*n* = 4) in ABTS, DPPH, CUPRAC, and Folin assays and in triplicate (*n* = 3) in the ORAC assay. For the different antioxidant assays, values were obtained using at least two dilution factors. Significant differences among the mean values when extraction was performed using different solvents (and when antioxidant capacity was measured using the same methodology for different samples) were assessed via a one-way ANOVA test (95% confidence level, GraphPad Prism software, version 9.5.0).

## 3. Results and Discussion

### 3.1. Selection of the Solid–liquid Extraction Solvent

Olive leaves from sampling spot #1 (sample 1, Table S1) were used as a model to evaluate the influence of the organic solvent during the extraction of gallic acid, hydroxytyrosol, catechin, oleuropein, pinoresinol, caffeic acid, verbascoside, rutin, and luteolin. Quercetin was not assessed during this study. These compounds were selected for monitoring due to their interesting features for food product incorporation, such as their antioxidant potential along with other claimed health benefits (e.g., anti-tumoral, hypoglycaemic, anti-cholesterolemic effects, etc.) [17].

**Figure 1:**
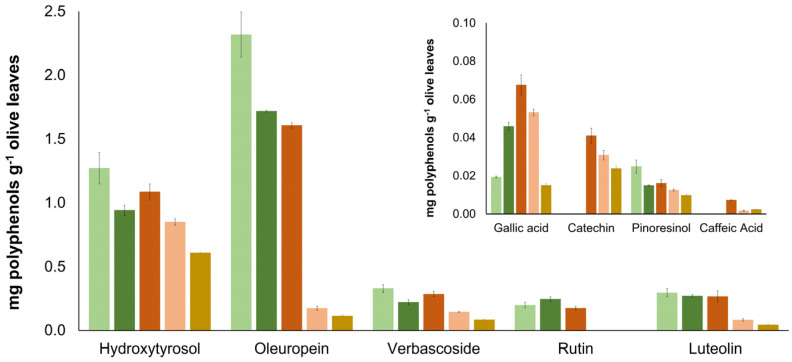
The quantity (mg) of polyphenolic compounds found in 1 g of olive leaves from sample 1 when extraction was performed using 80% (*v*/*v*) MeOH (light green), 80% (*v*/*v*) EtOH (dark green), 50% (*v*/*v*) EtOH (dark orange), 10% (*v*/*v*) EtOH (light orange) and 100% water (brown) each containing 0.1% (*v*/*v*) of formic acid. The values for gallic acid, catechin, pinoresinol, and caffeic acid are depicted as an insert at a reduced scale. Quercetin was not assessed during this study.

From these, oleuropein >> hydroxytyrosol > verbascoside, luteolin, and rutin were the major compounds found in the leaves (Figure 1). Regarding oleuropein, extraction was more efficient for 80% (*v*/*v*) MeOH, with 2.3 ± 0.2 mg being extracted per g of the leaves, followed by 80% (*v*/*v*) EtOH and 50% (*v*/*v*) EtOH (ANOVA, *p* < 0.0001), with 1.72 ± 0.01 and 1.61 ± 0.02 mg g^−1^ of leaves, respectively. Hydroxytyrosol’s (log P = 0.89) best extraction yields were accomplished using 80% (*v*/*v*) MeOH or 50% (*v*/*v*) EtOH as the extraction solvents, with 1.3 ± 0.1 and 1.1 ± 0.1 mg g^−1^ of leaves (Figure 1). Likewise, the best extraction yields for verbascoside were accomplished for extraction solvents containing 80% (*v*/*v*) MeOH or 50% (*v*/*v*) EtOH, with no differences obtained between the two solvents (ANOVA, *p* < 0.0001). For luteolin and rutin, no significant differences were also attained between the extraction yields for 80% (*v*/*v*) MeOH and 50% (*v*/*v*) EtOH. 

Considering the obtained results, the use of extraction solvents containing ≥50% organic solvent resulted in higher extraction yields, with significative losses obtained for lower contents of the organic solvent for hydroxytyrosol, oleuropein, verbascoside and luteolin (with losses up to 33, 92, 56 and 86%, respectively).

Thus, as the incorporation of the extracted compounds in food products was intended, and considering the extraction yields attained, 50% (*v*/*v*) of the EtOH solution was selected as the extraction solvent as a compromise between compound extraction yields and a greener and safer profile in comparison to the use of MeOH. 

### 3.2. Sample Analysis

#### 3.2.1. Quantification of Polyphenols

The type and amount of phenolic compounds found in olive leaves can vary depending on a number of factors, including solar exposure, the nutrient composition of the soil, the age and cultivar of the olive tree, climate conditions, geographic location, and agricultural practices [17]. Therefore, towards the evaluation of the impact of polyphenol content on antioxidant capacity values, twelve samples from distinct locations in Portugal (Table S1) were analyzed using the extraction solvent selected previously. The samples presented different quantities and types of polyphenols in their composition (Table 1). Chromatograms from a standard solution and from sample 5 are presented in Figure S1. All samples had hydroxytyrosol, catechin, rutin, oleuropein, luteolin, verbascoside, and quercetin (Table 1). Sample 12 was the only sample not presenting pinoresinol. Samples 1–6 and 8 also presented gallic acid in their composition. Caffeic acid was only present in 8 of the tested samples (samples 2, 3, 5, 6, 7, 8, 9, 11).

Oleuropein was the major compound found in the leaves, followed by verbascoside, hydroxytyrosol, luteolin, and rutin. Gallic acid and caffeic acid were the less representative polyphenols.

Sample 5 presented the highest levels of oleuropein and caffeic acid (≈29 and 0.1 mg g^−1^ olive leaves) and was the second sample with higher levels of hydroxytyrosol, verbascoside, and pinoresinol (≈0.9, 1.5, 0.2 mg g^−1^ leaves, respectively). This sample presented all the polyphenols under quantification and held the highest % of polyphenols per g^−1^ of leaves (3.26%, *w*/*w*).

Likewise, sample 7 was second with the highest quantity of total polyphenols (2.30%, *w*/*w*). This sample presented the highest levels of verbascoside (1.8 ± 0.2 mg g^−1^ leaves) and was the second sample with the highest amounts of oleuropein (19 ± 1 mg g^−1^ leaves), rutin (0.90 ± 0.07 mg g^−1^ leaves), and caffeic acid (0.08 ± 0.01 mg g^−1^ leaves).

Sample 8 showed the highest levels of hydroxytyrosol (1.5 ± 0.1 mg g^−1^ leaves) and rutin (1.0 ± 0.1 mg g^−1^ leaves) and was the second sample with the highest levels of quercetin (0.3 ± 0.01 mg g^−1^ leaves). This sample presented 0.91% (*w*/*w*) of polyphenols.

Thus, samples 5, 7, and 8 were among the most interesting due to their higher contents of polyphenols, such as hydroxytyrosol, oleuropein, verbascoside and/or rutin and quercetin. These were also within the top 4 samples with the highest quantities of total polyphenols per g^−1^ of the leaves (sample 5 > sample 7 > sample 9 > sample 8). Most likely, the increased content of polyphenols found for these samples did not result from geographic location and climate but rather from other factors, such as olive tree variety (cultivar), and/or age, soil composition, and/or agricultural practices. For example, samples 7 and 8 were collected in places closer to where samples 11 and 12 were gathered (Table S1). Nevertheless, the polyphenolic content from samples 7 and 8 greatly varied from those found in samples 11 and 12, lessening the importance of geographical location for these samples.

#### 3.2.2. Antioxidant Activity

The antioxidant capacity of each sample was assessed using different antioxidant capacity assays (Table 2). Folin, CUPRAC, ABTS, and DPPH antioxidant capacity assays are based on the transference of an electron from the antioxidant species to the spectrophotometric probe/chromogenic reagent (in the case of Folin and CUPRAC) or to the colored radical reagent (in this case, ABTS and DPPH) [43].

Samples 2, 5, 7, and 11 were in the top 4 samples with the highest antioxidant capacity values for these assays. From these, sample 5, which holds the highest quantity of total polyphenols (Table 1), presented significantly higher antioxidant capacity values (*p* ˂ 0.05, one-way ANOVA) in comparison to the other 3 samples (Figure S2). From this top 4, sample 7, the second sample with the highest quantity of total polyphenols, only exhibited significantly higher antioxidant capacity values in relation to sample 2 and sample 11 for the ABTS assay (*p* = 0.0392, one-way ANOVA). 

Although the results for sample 5 are aligned with the higher quantity of polyphenols amongst all the samples (32.6 mg per g^−1^ olive leaves), the determined antioxidant capacity values do not linearly correlate in relation to other samples with the total quantity of polyphenols; in fact, despite sample 5 holding a total amount of polyphenols ca. 4 times higher than sample 2, this last only presented a slight lower antioxidant capacity for the Folin, CUPRAC, ABTS and DPPH assays.

The ORAC method belongs to the group of assays measuring antioxidant protection from oxidation via biologically relevant radicals through a sequential proton-loss electron-transfer mechanism [42,46,49]. In this assay, there is a constant generation of radicals that are tackled by the antioxidant species until its consumption [46]. After antioxidant species depletion, the oxidation of the fluorescent probe by the radicals occurs, with consequent photobleaching. The assessed antioxidant capacity values using the ORAC methodology reflect not only the stoichiometry of antioxidant compounds but also their reactivity, including their kinetic profile, in opposition to the other antioxidant methodologies used in this work. Moreover, antioxidants’ reaction with the radicals may lead to the formation of by-products with antioxidant capacity, which also impacts the measured values [46].

In fact, for ORAC assays, the total quantity of polyphenols showed less relevance for the prediction of the antioxidant capacity. In this assay, samples 5 and 7 did not stand out with the higher antioxidant capacity values despite their markedly higher quantity of total polyphenols, with samples 4 and 8 exhibiting the highest antioxidant capacity values for the ORAC assay.

Sample 4 presented higher contents of pinoresinol, rutin, quercetin, luteolin, and verbascoside in comparison to sample 5 (Table 1). Likewise, sample 8 presented higher contents of hydroxytyrosol, catechin, rutin, quercetin, and luteolin in comparison to sample 5. These differences may justify the obtained results. Indeed, different compounds hold distinct antioxidant capacity values according to the antioxidant capacity assay methodology, as clearly demonstrated when the TEAC values for each standard compound were assessed using the different antioxidant capacity assays (Table S3). In addition, in the case of ORAC, differences in relation to the values assessed by Folin, CUPRAC, ABTS, and DPPH assays are expected [42] due to the different antioxidant capacity mechanisms taking place in the first in relation to these assays but also due to the importance of antioxidant kinetic aspects and formation of antioxidant by-products for this methodology [46]. 

**Figure 2:**
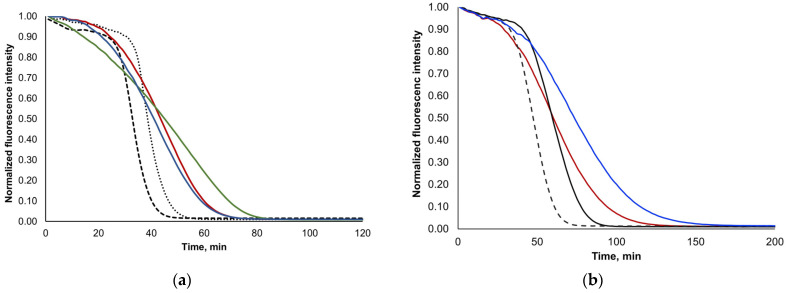
ORAC curves obtained for (**a**) the standard antioxidant compounds oleuropein (10 μM, dashed black line), hydroxytyrosol (8 μM, dotted black line), rutin (10 μM, solid red line), luteolin (10 μM, solid blue line) and quercetin (9 μM, solid green line) and for (**b**) samples 4 (solid red line), 5 (solid black line), 7 (dashed black line) and 8 (solid blue line) analyzed at the same dilution.

In fact, for the ORAC assay, quercetin was already described as a slow-reacting antioxidant compound [46], leading to the absence of a lag phase (the time of protection of fluorescein fluorescence decay given by the antioxidant species in relation to the fast reaction of the antioxidant species with the AAPH-generated radicals) in the ORAC assay curve and to a tailed decay of the fluorescence probe signal (related with slow-acting antioxidant mechanism). A similar ORAC profile to that of quercetin (Figure 2a) was found for samples 4 and 8 (Figure 2b). Moreover, ORAC profiles similar to that of quercetin were found for rutin and luteolin, indicating that these are also compounds with a slow-reacting antioxidant mechanism (Figure 2a). Opposite ORAC profiles were obtained for oleuropein and hydroxytyrosol (Figure 2a), which presented a clearly defined lag phase followed by faster fluorescein photobleaching. Therefore, the increased content of these compounds (quercetin, luteolin, and rutin) in samples 4 and 8 may justify the higher antioxidant capacity values for these samples under the ORAC methodology in comparison to samples 5 and 7. In fact, samples 4 and 8 presented ORAC curve profiles with shorter lag phases and with a more tailed decay of fluorescent probe signals [46] in relation to these (Figure 2b).

Subsequently, to attempt to elucidate the predictability of the antioxidant capacity for each methodology according to the polyphenolic content, the TEAC value found for each compound was multiplied by the concentration of the compound on the olive leaf samples to determine the theoretical TEAC value (details on Section 2—materials and methods). The theoretical TEAC value was then compared to the value determined experimentally for each sample across the different antioxidant methodologies to exploit the correlations between polyphenolic quantity and TEAC values with the antioxidant capacity, as well as to evaluate possible synergetic antioxidant effects. All theoretical TEAC values, calculated by the sum of individual contributions of the assessed polyphenols, were found to be lower than the values determined experimentally. This was expected, considering that not all compounds present in the olive leaves are quantified. Additionally, the presence of different antioxidant compounds may foster a synergetic action, where slow-reacting compounds can regenerate the antioxidant capacity of other sample components [46].

The total theoretical TEAC values determined for samples 4 and 8 were lower than those found for sample 5 and for all the antioxidant capacity assays. Indeed, these samples exhibited the most marked differences between theoretical and experimental TEAC values for the ORAC assay (experimental values 9 to 16× higher than theoretical values). Therefore, the increased antioxidant protection of samples 4 and 8 under the ORAC methodology was not possible to predict based on the TEAC values of individual compounds and their concentration. This emphasizes the importance of understanding the chemistry underlying each polyphenolic compound’s antioxidant mechanism and how it affects the overall afforded antioxidant protection. Therefore, these results show the importance of performing antioxidant studies along with the quantitative analysis of polyphenols when selecting samples for the further application of food enrichment purposes. For instance, sample 5 was found to be the most relevant in Section 3.2.1 to obtain a higher quantity of total polyphenolic compounds for future incorporation into food products (Table 1). This sample also presented higher antioxidant capacity values in assays where reducing and radical scavenging electron-transfer mechanisms were involved (Table 2). However, in more complex environments in which the antioxidant capacity was measured against biologically relevant radicals and in which the formation of by-products with antioxidant activity could occur, samples 4 and 8, which presented higher contents of slow-reacting antioxidants, revealed interesting antioxidant capacities. In fact, different olive leaf polyphenols hold different O-H dissociation proton affinity and electron-transfer enthalpies that differently impact the antioxidant protection mechanisms and, consequently, the antioxidant capacity measured by different methodologies [49,50,51]. Consequently, these aspects must be taken into consideration when selecting the extracts for food fortification, according to the aimed purposes, as some compounds may be more relevant for food stability purposes throughout storage while others may result in a higher antioxidant capacity in biological media.

### 3.3. Stability of Lyophilization Process and Bioaccessibility Studies

Considering the future incorporation of the extracted compounds in food products, the liquid extract from sample 5, which previously showed the highest content of total polyphenols, the highest amount of oleuropein, and the highest antioxidant activity using Folin, CUPRAC, ABTS, and DPPH, was converted to powder via lyophilization. A 25 mL ethanolic extract was prepared using 0.5 g of the dried olive leaves, resulting in ≈110 mg of powder, which represents ca. 20% of the mass of leaves used. Similar results were attained for sample 7, which presented the second-highest quantity of total polyphenols and for which 149 mg of the powder was recovered. The content of the dried extracts in hydroxytyrosol, oleuropein, pinoresinol, verbascoside, rutin, quercetin, and luteolin was assessed (Table 3), with higher quantities of polyphenols’ g^−1^ dried extract being obtained in relation to those described in the literature [16]. Oleuropein represented 84% and 14% of the mass of the dried extract obtained from samples 5 and 7, respectively.

Nevertheless, the lyophilization of the liquid extracts impacted the quantity of polyphenols, with decreases in the content of polyphenols detected for all the assessed compounds (Table S4) in both samples. Thus, the optimization of the lyophilization procedure used herein may be required when envisioning an industrial application. 

**Figure 3:**
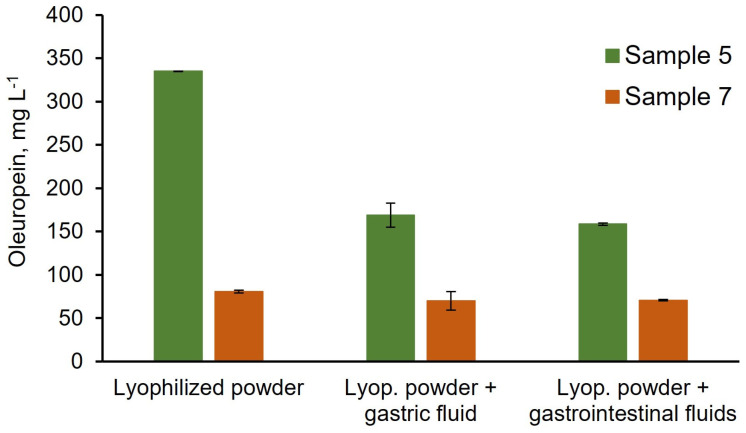
Concentration (mg L^−1^) of oleuropein found in the lyophilized powder prior to incubation with gastrointestinal surrogate fluids (left panel) vs. the concentration found upon incubation with (i) gastric fluid (middle panel) and (ii) gastric fluid followed by intestinal fluid (right panel) for samples 5 and 7.

Since the incorporation of olive leaf-extracted polyphenols in food products is a future aim, the bioaccessibility of oleuropein, the major compound present in the lyophilized powder, was assessed. These assays enable the portion of oleuropein capable of release from the powder for intestinal absorption to be unveiled [16]. For this, lyophilized powder was incubated with surrogate gastric fluid (pH 1.2) containing pepsin for 2 h at 37 °C. Likewise, analysis, when the powder was subsequently submitted to incubation with intestinal surrogate fluid (pH = 6.8, containing pancreatin, 4 h at 37 °C), was also performed. For sample 5, ca. 50% of the oleuropein present in the powder was found in the gastric surrogate fluid (Figure 3). The subsequent incubation with intestinal surrogate fluid did not markedly alter the content of determined oleuropein. Interestingly, for sample 7, ca. 87% of the oleuropein present in the lyophilized powder was found in the gastric surrogate fluid (Figure 3). As for sample 5, the further incubation of the powder with intestinal fluid did not markedly affect the content of oleuropein.

These results suggest that contact with the gastric fluid is detrimental to oleuropein release from the powder, as no marked differences were observed between the determined oleuropein levels after gastric-simulated digestion and after gastric+intestinal-simulated digestion for both samples. Likewise, the stability of oleuropein to intestinal surrogate fluid is also suggested by these results. Moreover, the amount of oleuropein released from the matrix upon simulated gastric digestion differed between samples 5 and 7. This could be related to different oleuropein release kinetics from the powder due to the differences in samples 5 and 7 powder composition [52]. Another possible justification would be that samples 5 and 7 contain different amounts of oleuropein isomers, as different bioaccessibility values have been reported for different oleuropein isomers [16].

## 4. Conclusions

In this work, the amount of 10 polyphenolic compounds (gallic acid, hydroxytyrosol, catechin, oleuropein, pinoresinol, caffeic acid, rutin, quercetin, luteolin, and verbascoside) present in olive leaves from 12 different Portuguese locations up north was assessed using HPLC-UV-Vis. The solvent selected for the extraction of these compounds was shown to impact the contents of hydroxytyrosol, oleuropein, verbascoside, and luteolin, with extraction solvents containing ≥50% of the organic solvent resulting in higher extraction yields for these compounds. Selecting a 50% (*v*/*v*) ethanolic solution was shown to be a good compromise regarding polyphenolic compound extraction yields and method adequacy for future food fortification with the extracted compounds. Besides the quantitative assessment of polyphenolic compounds in the different samples, the antioxidant capacity assessment in these samples was performed using different methodologies, considering that these provide different information regarding antioxidant mechanisms and capacity. While these assays corroborated the importance of total polyphenols for the antioxidant capacity, as measured by Folin, CUPRAC, ABTS, and DPPH assays, the results obtained via the ORAC methodology emphasized the importance of compounds bearing slow-reacting antioxidant mechanisms and yielding the formation of by-products with an antioxidant capacity in media resembling biological environments. In addition, the feasibility of extracted compounds for lyophilization and intestinal absorption was assessed, highlighting important features as well as optimization requirements when leveraging the potential of olive leaf waste for food fortification. Future work in this field is required to evaluate the impact of extracted compounds on increasing food stability throughout storage and/or for providing insights into the improved biological activities provided by the extracted compounds towards the development of functional foods. Additionally, the incorporation of olive leaf-extracted compounds into specific food processes, such as emulsification and encapsulation, requires further investigation.

## Figures and Tables

**Table 1:** Mass of polyphenols (mg per 100 g of leaves) found in the liquid extracts of the different samples of olive leaves under analysis.

	S1	S2	S3	S4	S5	S6	S7	S8	S9	S10	S11	S12
*Gallic Acid*	3.2 ± 0.1	1.6 ± 0.2	2.03 ± 0.05	2.2 ± 0.3	3.3 ± 0.1	3.57 ± 0.05	n/d	0.5 ± 0.1	n/d	n/d	n/d	n/d
*Hydroxytyrosol*	22 ± 3	25.1 ± 0.3	27 ± 2	21 ± 2	86 ± 8	56 ± 6	70 ± 5	146 ± 12	62 ± 8	49 ± 4	84 ± 3	13 ± 1
*Catechin*	1.9 ± 0.1	4.8 ± 0.2	7 ± 1	9 ± 1	4.1 ± 0.4	6 ± 1	22 ± 2	25.48 ± 0.01	3.7 ± 0.3	4.4 ± 0.1	15.5 ± 0.1	8.1 ± 0.5
*Oleuropein*	164 ± 22	583 ± 15	131 ± 8	71 ± 6	2894 ± 214	103 ± 9	1852 ± 141	474 ± 56	1040 ± 131	135 ± 17	518 ± 40	165 ± 12
*Pinoresinol*	8.9 ± 0.4	5.3 ± 0.7	3.4 ± 0.1	29 ± 4	22 ± 2	10.1 ± 0.1	8 ± 1	6 ± 1	7.4 ± 0.2	1.8 ± 0.1	3.3 ± 0.2	n/d
*Caffeic Acid*	n/d	3.04 ± 0.02	2.72 ± 0.01	n/d	9 ± 1	0.39 ± 0.01	8 ± 1	0.6 ± 0.1	2.1 ± 0.2	n/d	0.58 ± 0.05	n/d
*Rutin*	12 ± 2	35 ± 3	52.6 ± 0.2	63 ± 3	52 ± 5	18 ± 1	90 ± 7	101 ± 12	31 ± 4	17.3 ± 2.1	67 ± 6	20 ± 2
*Quercetin*	3.2 ± 0.2	12.6 ± 0.3	10 ± 1	35 ± 3	12 ± 2	10 ± 1	19 ± 2	26 ± 2	10 ± 1	10.2 ± 1.1	17 ± 2	3.8 ± 0.4
*Luteolin*	32 ± 2	70 ± 2	78 ± 3	96 ± 6	25 ± 1	40 ± 2	58 ± 8	63 ± 4	28 ± 2	30 ± 3	62 ± 5	6 ± 1
*Verbascoside*	14 ± 2	13 ± 1	7.8 ± 0.4	52 ± 3	150 ± 13	106 ± 11	183 ± 21	67 ± 7	66 ± 7	67 ± 7	77 ± 5	3.3 ± 0.1

n/d, not determined.

**Table 2:** Antioxidant capacity values determined for the extracts of the different samples under analysis using each methodology.

	FOLIN ^a^	DPPH ^b^	ABTS ^b^	CUPRAC ^b^	ORAC ^b^
S1	0.18 ± 0.03	0.11 ± 0.01	0.28 ± 0.03	0.30 ± 0.01	0.9 ± 0.1
S2	0.31 ± 0.05	0.17 ± 0.02	0.37 ± 0.05	0.43 ± 0.06	0.22 ± 0.04
S3	0.24 ± 0.03	0.14 ± 0.02	0.36 ± 0.04	0.31 ± 0.02	0.53 ± 0.09
S4	0.22 ± 0.01	0.09 ± 0.01	0.25 ± 0.04	0.27 ± 0.01	1.4 ± 0.2
S5	0.38 ± 0.05	0.19 ± 0.01	0.51 ± 0.05	0.52 ± 0.04	0.9 ± 0.1
S6	0.15 ± 0.01	0.06 ± 0.01	0.19 ± 0.02	0.22 ± 0.01	1.0 ± 0.1
S7	0.33 ± 0.04	0.16 ± 0.02	0.46 ± 0.02	0.43 ± 0.02	0.61 ± 0.07
S8	0.22 ± 0.01	0.12 ± 0.01	0.31 ± 0.04	0.36 ± 0.02	1.8 ± 0.1
S9	0.28 ± 0.01	0.15 ± 0.01	0.35 ± 0.02	0.35 ± 0.01	0.78 ± 0.08
S10	0.18 ± 0.02	0.16 ± 0.02	0.23 ± 0.02	0.22 ± 0.01	0.25 ± 0.05
S11	0.31 ± 0.04	0.16 ± 0.01	0.41 ± 0.04	0.42 ± 0.02	0.9 ± 0.1
S12	0.18 ± 0.01	0.142 ± 0.004	0.21 ± 0.01	0.25 ± 0.01	0.27 ± 0.01

^a^ Values expressed as mmol of gallic acid g^−1^ sample. ^b^ Values expressed as mmol Trolox g^−1^ sample.

**Table 3:** Quantity of polyphenolic compounds (mg) found per g of the dried extract.

	Sample 5	Sample 7
Hydroxytyrosol	14.4 ± 0.1	5.5 ± 0.3
Oleuropein	837 ± 1	135 ± 3
Pinoresinol	7.2 ± 0.1	1.93 ± 0.2
Verbascoside	21.7 ± 0.1	6.4 ± 0.1
Rutin	15.1± 0.1	8.2± 0.3
Quercetin	3.30 ± 0.01	2.0 ± 0.1
Luteolin	4.1 ± 0.1	4.9 ± 0.1

## Data Availability

Data are contained within the article and supplementary materials.

## Supplementary Materials

The following supporting information can be downloaded at: https://www.mdpi.com/article/10.3390/foods13020189/s1, Figure S1. Chromatograms at (a) 280 nm, (b) 320 nm and (c) 350 nm depicting: a standard solution containing 10 mg L^−1^ of the following compounds (red line): (1) gallic acid, (2) hydroxytyrosol, (3) catechin, (4) caffeic acid, (5) verbascoside, (6) rutin, (7) oleuropein, (8) pinoresinol, (9) quercetin, and (10) luteolin; 50% (*v*/*v*) EtOH extract from sample 5, undiluted (dark green line) and diluted 2× (light green line) and, for the same extract, diluted 2× supplemented with 5 mg L^−1^ of each compound (orange line). Compounds 1–3, 7, 8 were monitored at 280 nm; compounds 4 and 5 were monitored at 320 nm; compounds 6, 9, 10 were monitored at 350 nm. The grey line represents the injection of mobile phase; Figure S2: Antioxidant (AOX) capacity values (mean ± SD) for samples 2, 5, 7 and 11 as determined by Folin, DPPH, ABTS and CUPRAC methods; Table S1: Geographical locations of olive leaf samples collection; Table S2: HPLC calibration curves for standard compounds; Table S3: TEAC values determined by each methodology for the polyphenolic compounds under analysis; Table S4: Content of polyphenols (mg) per g of olive leaves determined in liquid extracts before and upon lyophilization.

## References

[1] De Bruno A., Romeo R., Piscopo A., Poiana M. (2021). Antioxidant quantification in different portions obtained during olive oil extraction process in an olive oil press mill. J. Sci. Food Agric..

[2] Abdallah M., Marzocco S., Adesso S., Zarrouk M., Guerfel M. (2018). Olive oil polyphenols extracts inhibit inflammatory markers in J774A.1 murine macrophages and scavenge free radicals. Acta Histochem..

[3] Bozzetto L., Alderisio A., Clemente G., Giorgini M., Barone F., Griffo E., Costabile G., Vetrani C., Cipriano P., Giacco A. (2019). Gastrointestinal effects of extra-virgin olive oil associated with lower postprandial glycemia in type 1 diabetes. Clin. Nutr..

[4] Bilal R.M., Liu C., Zhao H., Wang Y., Farag M.R., Alagawany M., Hassan F.-u., Elnesr S.S., Elwan H.A., Qiu H. (2021). Olive oil: Nutritional applications, beneficial health aspects and its prospective application in poultry production. Front. Pharmacol..

[5] Kaddoumi A., Denney T.S., Deshpande G., Robinson J.L., Beyers R.J., Redden D.T., Praticò D., Kyriakides T.C., Lu B.N., Kirby A.N. (2022). Extra-Virgin Olive Oil Enhances the Blood-Brain Barrier Function in Mild Cognitive Impairment: A Randomized Controlled Trial. Nutrients.

[6] Amel N., Wafa T., Samia D., Yousra B., Issam C., Cheraif I., Attia N., Mohamed H. (2016). Extra virgin olive oil modulates brain docosahexaenoic acid level and oxidative damage caused by 2,4-Dichlorophenoxyacetic acid in rats. J. Food Sci. Technol.-Mysore.

[7] European Commission Olives, the Source of “Liquid Gold,” Offer More Riches to Unlock. https://ec.europa.eu/research-and-innovation/en/horizon-magazine/olives-source-liquid-gold-offer-more-riches-unlock.

[8] Selim S., Albqmi M., Al-Sanea M.M., Alnusaire T.S., Almuhayawi M.S., AbdElgawad H., Al Jaouni S.K., Elkelish A., Hussein S., Warrad M. (2022). Valorizing the usage of olive leaves, bioactive compounds, biological activities, and food applications: A comprehensive review. Front. Nutr..

[9] European Commission Olive Oil in the EU. https://agriculture.ec.europa.eu/farming/crop-productions-and-plant-based-products/olive-oil_en.

[10] Abbattista R., Ventura G., Calvano C.D., Cataldi T.R., Losito I. (2021). Bioactive compounds in waste by-products from olive oil production: Applications and structural characterization by mass spectrometry techniques. Foods.

[11] Teatro Naturale Il Portogallo Olivicolo è il Paese Emergente nell’Europa dell’olio di Oliva. https://www.teatronaturale.it/tracce/mondo/36891-il-portogallo-olivicolo-e-forma-emergente-nell-europa-dell-olio-di-oliva.htm.

[12] Olive Oil Times Portugal May Be the Third-Largest Olive Oil Producer by 2030. https://www.oliveoiltimes.com/production/portugal-may-be-the-third-largest-olive-oil-producer-by-2030/74445.

[13] Tapia-Quirós P., Montenegro-Landívar M.F., Reig M., Vecino X., Cortina J.L., Saurina J., Granados M. (2022). Recovery of polyphenols from agri-food by-products: The olive oil and winery industries cases. Foods.

[14] Olive Oil Times Record Yields for Portugal in the 2021/22 Crop Year. https://www.oliveoiltimes.com/production/record-yields-for-portugal/105753.

[15] Azaizeh H., Abu Tayeh H.N., Gerchman Y., Krishnaraj Rathinam N., Sani R.K. (2020). Chapter 2—Valorisation of olive oil industry solid waste and production of ethanol and high value-added biomolecules. Biovalorisation of Wastes to Renewable Chemicals and Biofuels.

[16] Duque-Soto C., Quirantes-Piné R., Borrás-Linares I., Segura-Carretero A., Lozano-Sánchez J. (2022). Characterization and influence of static in vitro digestion on bioaccessibility of bioactive polyphenols from an olive leaf extract. Foods.

[17] Rahmanian N., Jafari S.M., Wani T.A. (2015). Bioactive profile, dehydration, extraction and application of the bioactive components of olive leaves. Trends Food Sci. Technol..

[18] Zhang C.C., Xin X.T., Zhang J.M., Zhu S.L., Niu E.L., Zhou Z.J., Liu D.Q. (2022). Comparative evaluation of the phytochemical profiles and antioxidant potentials of olive leaves from 32 cultivars grown in China. Molecules.

[19] Mansour H.M.M., Zeitoun A.A., Abd-Rabou H.S., El Enshasy H.A., Dailin D.J., Zeitoun M.A.A., El-Sohaimy S.A. (2023). Antioxidant and anti-diabetic properties of olive (*Olea europaea*) leaf extracts: In vitro and in vivo evaluation. Antioxidants.

[20] Romero-Márquez J.M., Navarro-Hortal M.D., Forbes-Hernández T.Y., Varela-López A., Puentes J.G., Del Pino-García R., Sánchez-González C., Elio I., Battino M., García R. (2023). Exploring the antioxidant, neuroprotective, and anti-inflammatory potential of olive leaf extracts from Spain, Portugal, Greece, and Italy. Antioxidants.

[21] Sanchez-Gutiérrez M., Bascón-Villegas I., Rodríguez A., Pérez-Rodríguez F., Fernández-Prior A., Rosal A., Carrasco E. (2021). Valorisation of *Olea europaea* L. olive leaves through the evaluation of their extracts: Antioxidant and antimicrobial Activity. Foods.

[22] Gandhi G.R., Vasconcelos A.B.S., Wu D.T., Li H.B., Antony P.J., Li H., Geng F., Gurgel R.Q., Narain N., Gan R.Y. (2020). Citrus flavonoids as promising phytochemicals targeting diabetes and related complications: A systematic review of in vitro and in vivo studies. Nutrients.

[23] Pirkovic A., Vilotic A., Borozan S., Nacka-Aleksic M., Bojic-Trbojevic Ä., Krivokuca M.J., Battino M., Giampieri F., Dekanski D. (2023). Oleuropein Attenuates Oxidative Stress in Human Trophoblast Cells. Antioxidants.

[24] Pojero F., Aiello A., Gervasi F., Caruso C., Ligotti M.E., Calabrò A., Procopio A., Candore G., Accardi G., Allegra M. (2023). Effects of Oleuropein and Hydroxytyrosol on Inflammatory Mediators: Consequences on Inflammaging. Int. J. Mol. Sci..

[25] Subias-Gusils A., Alvarez-Monell A., Boqué N., Caimari A., Mariné-Casadó R., Escorihuela R.M., Solanas M. (2023). Effects of a Calorie-Restricted Cafeteria Diet and Oleuropein Supplementation on Adiposity and mRNA Expression of Energy Balance Related Genes in Obese Male Rats. Metabolites.

[26] Carnevale R., Silvestri R., Loffredo L., Novo M., Cammisotto V., Castellani V., Bartimoccia S., Nocella C., Violi F. (2018). Oleuropein, a component of extra virgin olive oil, lowers postprandial glycaemia in healthy subjects. Br. J. Clin. Pharmacol..

[27] Vezza T., Rodríguez-Nogales A., Algieri F., Garrido-Mesa J., Romero M., Sánchez M., Toral M., Martín-García B., Gómez-Caravaca A.M., Arráez-Román D. (2019). The metabolic and vascular protective effects of olive (*Olea europaea* L.) leaf extract in diet-induced obesity in mice are related to the amelioration of gut microbiota dysbiosis and to its immunomodulatory properties. Pharmacol. Res..

[28] Andújar-Tenorio N., Cobo A., Martínez-Rodríguez A.M., Hidalgo M., Prieto I., Gálvez A., Martínez-Cañamero M. (2023). Intestinal microbiota modulation at the strain level by the olive oil polyphenols in the diet. Front. Nutr..

[29] Bulotta S., Celano M., Lepore S.M., Montalcini T., Pujia A., Russo D. (2014). Beneficial effects of the olive oil phenolic components oleuropein and hydroxytyrosol: Focus on protection against cardiovascular and metabolic diseases. J. Transl. Med..

[30] Sciandra F., Bottoni P., De Leo M., Braca A., Brancaccio A., Bozzi M. (2023). Verbascoside elicits its beneficial effects by enhancing mitochondrial spare respiratory capacity and the Nrf2/HO-1 mediated antioxidant system in a murine skeletal muscle cell line. Int. J. Mol. Sci..

[31] Mir-Cerdà A., Nuñez O., Granados M., Sentellas S., Saurina J. (2023). An overview of the extraction and characterization of bioactive phenolic compounds from agri-food waste within the framework of circular bioeconomy. TrAC-Trends Anal. Chem..

[32] Monteleone J.I., Sperlinga E., Siracusa L., Spagna G., Parafati L., Todaro A., Palmeri R. (2021). Water as a solvent of election for obtaining oleuropein-rich extracts from olive (*Olea europaea*) leaves. Agronomy.

[33] da Rosa G.S., Vanga S.K., Gariepy Y., Raghavan V. (2019). Comparison of microwave, ultrasonic and conventional techniques for extraction of bioactive compounds from olive leaves (*Olea europaea* L.). Innov. Food Sci. Emerg. Technol..

[34] Lama-Munoz A., Contreras M.D.M., Espinola F., Moya M., Romero I., Castro E. (2020). Content of phenolic compounds and mannitol in olive leaves extracts from six Spanish cultivars: Extraction with the Soxhlet method and pressurized liquids. Food Chem..

[35] Caballero A.S., Romero-García J.M., Castro E., Cardona C.A. (2020). Supercritical fluid extraction for enhancing polyphenolic compounds production from olive waste extracts. J. Chem. Technol. Biotechnol..

[36] Yusoff I.M., Taher Z.M., Rahmat Z., Chua L.S. (2022). A review of ultrasound-assisted extraction for plant bioactive compounds: Phenolics, flavonoids, thymols, saponins and proteins. Food Res. Int..

[37] Akli H., Grigorakis S., Kellil A., Loupassaki S., Makris D.P., Calokerinos A., Mati A., Lydakis-Simantiris N. (2022). Extraction of Polyphenols from Olive Leaves Employing Deep Eutectic Solvents: The Application of Chemometrics to a Quantitative Study on Antioxidant Compounds. Appl. Sci..

[38] Jaski J.M., da Cruz R.M.S., Pimentel T.C., Stevanato N., da Silva C., Barao C.E., Cardozo L. (2023). Simultaneous Extraction of Bioactive Compounds from *Olea europaea* L. Leaves and Healthy Seed Oils Using Pressurized Propane. Foods.

[39] Vasilopoulou K., Papadopoulos G.A., Lioliopoulou S., Pyrka I., Nenadis N., Savvidou S., Symeon G., Dotas V., Panitsidis I., Arsenos G. (2023). Effects of Dietary Supplementation of a Resin-Purified Aqueous-Isopropanol Olive Leaf Extract on Meat and Liver Antioxidant Parameters in Broilers. Antioxidants.

[40] Clodoveo M.L., Crupi P., Annunziato A., Corbo F. (2022). Innovative Extraction Technologies for Development of Functional Ingredients Based on Polyphenols from Olive Leaves. Foods.

[41] U.S. Pharmacopeia (2015). U.S. Pharmacopeial Convention. Reagents, indicators, and solutions. Solutions. Buffer Solutions. United States Pharmacopeia and National Formulary (USP 38–NF 33).

[42] Magalhães L.M., Ramos I.I., Reis S., Segundo M.A. (2014). Antioxidant profile of commercial oenological tannins determined by multiple chemical assays. Aust. J. Grape Wine Res..

[43] Huang D., Ou B., Prior R.L. (2005). The chemistry behind antioxidant capacity assays. J. Agric. Food Chem..

[44] Brand-Williams W., Cuvelier M.-E., Berset C. (1995). Use of a free radical method to evaluate antioxidant activity. LWT-Food Sci. Technol..

[45] Magalhães L.M., Segundo M.A., Reis S., Lima J.L.F.C. (2008). Methodological aspects about in vitro evaluation of antioxidant properties. Anal. Chim. Acta.

[46] Carvalho J.R., Meireles A.N., Marques S.S., Gregório B.J., Ramos I.I., Silva E.M., Barreiros L., Segundo M.A. (2023). Exploiting kinetic features of ORAC assay for evaluation of radical scavenging capacity. Antioxidants.

[47] Marques S.S., Magalhaes L.M., Tóth I.V., Segundo M.A. (2014). Insights on antioxidant assays for biological samples based on the reduction of copper complexes-The importance of analytical conditions. Int. J. Mol. Sci..

[48] Baghalabadi V., Razmi H., Doucette A. (2021). Salt-mediated organic solvent precipitation for enhanced recovery of peptides generated by pepsin digestion. Proteomes.

[49] Zhang D., Liu Y.X., Chu L., Wei Y., Wang D., Cai S.B., Zhou F., Ji B.P. (2013). Relationship between the structures of flavonoids and Oxygen Radical Absorbance Capacity values: A quantum chemical analysis. J. Phys. Chem. A.

[50] Nenadis N., Wang L.F., Tsimidou M.Z., Zhang H.Y. (2005). Radical scavenging potential of phenolic compounds encountered in O. europaea products as indicated by calculation of bond dissociation enthalpy and ionization potential values. J. Agric. Food Chem..

[51] Biela M., Rimarcík J., Senajová E., Kleinová A., Klein E. (2020). Antioxidant action of deprotonated flavonoids: Thermodynamics of sequential proton-loss electron-transfer. Phytochemistry.

[52] Gregório B.J.R., Pereira A.M., Fernandes S.R., Matos E., Castanheira F., Almeida A.A., Fonseca A.J.M., Cabrita A.R.J., Segundo M.A. (2020). Flow-based dynamic approach to assess bioaccessible zinc in dry dog food samples. Molecules.

