# Supplementary material for: Olive Oil Waste as a Source of Functional Food Ingredients: Assessing Polyphenolic Content and Antioxidant Activity in Olive Leaves
Source: Foods. 2024 Jan 6;13(2):189. doi: 10.3390/foods13020189 (PMC10814828; doi:10.3390/foods13020189)
Supplement: Supplementary file 1 [file foods-13-00189-s001.zip › foods-2792455-supplementary.pdf]

Supporting Information

Olive OilWaste as a Source of Functional Food Ingredients:

Assessing Polyphenolic Content and Antioxidant Activity in

Olive Leaves

Carolina L. Ronca [1,2,3] , Sara S.Marques [1] , Alberto Ritieni [2,†] , Rafael Giménez-Martínez [3], Luisa Barreiros [1,4]

and Marcela A. Segundo [1,*]

[1] LAQV, REQUIMTE, Department of Chemical Sciences, Faculty of Pharmacy, University of Porto,

4099-002 Porto, Portugal; carolinaronca@correo.ugr.es (C.L.R.); scmarques@ff.up.pt (S.S.M.);

lbarreiros@ff.up.pt (L.B.)

[2] Department of Pharmacy, Faculty of Pharmacy, University of Naples "Federico II", 80138 Naples, Italy

[3] Department of Nutrition and Bromatology, School of Pharmacy, University of Granada, 18012 Granada, Spain;

rafaelg@ugr.es

[4] School of Health, Polytechnic Institute of Porto, 4200-072 Porto, Portugal

* Correspondence: msegundo@ff.up.pt; Tel.: +351-220428676

† Deceased author.

Table S1. Geographical locations of olive leaves samples collection.

Sample ID	City	GPS coordinates
**1**	Arcos de Valdevez	41° 51' 24.4" N, 8° 25' 46.7"W
**2**	Arcos de Valdevez	41° 51' 25.8"N, 8° 25' 46.9"W
**3**	Arcos de Valdevez	41° 51' 17.6"N, 8° 26' 09.9"W
**4**	Vila Nova de Gaia	41° 03' 52.1"N, 8° 32' 52."W
**5**	Braga	41° 32' 23,66"N, 8° 23' 34"W
**6**	Luso	40° 23' 15.2"N, 8° 23' 17.8"W
**7**	Vila Real	41° 15' 47"N, 7° 44' 5"W
**8**	Mirandela	41° 26' 23.8"N, 7° 17' 34.5"W
**9**	Cervães, Vila Verde	41°35'58.8"N 8°31'08.8"W
**10**	Porto	41° 08' 58" N, 8° 36' 39"W
**11**	Paços de Ferreira	41°14'40.1"N 8°24'12.8"W
**12**	Celorico de Basto	41°23'57.6"N 8°00'05.4"W

Table S2. HPLC calibration curves for standard compounds.

Standards	Slope (L^{-1} mg)	Intercept	R	R^2	LOD (mg L^{-1})[a]
**Gallic acid**	48595	-13528	0.998	0.996	0.07
**Hydroxytyrosol**	9724	-704	1.000	0.999	0.2
**Catechin**	11545	-1786	1.000	0.999	0.1
**Oleuropein**	4956	-1771	0.994	0.988	0.03
**Pinoresinol**	34957	-12450	0.997	0.994	0.01
**Caffeic acid**	98558	17293	1.000	1.000	0.01
**Rutin**	28909	6008	0.987	0.974	0.03
**Quercetin**	23356	-9533	1.000	0.999	0.2
**Luteolin**	45584	-3772	0.989	0.978	0.2

[a] calculated from the signal to noise ratio, n=10. LOQ values were < 0.25 mg L^{-1} for all tested compounds.

Table S3. TEAC values determined by each methodology for the polyphenolic compounds under analysis.

	Folin[a]	CUPRAC[b]	ABTS[b]	DPPH[b]	ORAC[b]
**Gallic Acid**	1.00[a]	2.0 ± 0.1	4.9 ± 0.1	5.3 ± 0.5	1.9 ± 0.1
**Hydroxytyrosol**	0.92 ± 0.01	1.60 ± 0.04	1.50 ± 0.01	0.99 ± 0.04	6 ± 1
**Catechin**	1.58 ± 0.01	2.39 ± 0.04	4.3 ± 0.1	3.3 ± 0.1	8.9 ± 0.6
**Caffeic Acid**	0.98 ± 0.01	1.11 ± 0.03	2.11 ± 0.02	1.04 ± 0.03	6.5 ± 0.8
**Rutin**	1.57 ± 0.01	1.9 ± 0.1	2.55 ± 0.03	2.1 ± 0.2	10.2 ± 0.8
**Oleuropein**	0.99 ± 0.01	2.07 ± 0.03	1.32 ± 0.02	0.99 ± 0.04	5.6 ± 0.2
**Pinoresinol**	1.19 ± 0.01	1.19 ± 0.03	2.6 ± 0.1	1.1 ± 0.1	8 ± 1
**Quercetin**	2.0 ± 0.1	1.64 ± 0.02	3.3 ± 0.1	2.4 ± 0.1	11 ± 2
**Luteolin**	2.71 ± 0.02	2.08 ± 0.04	3.43 ± 0.06	2.0 ± 0.1	9 ± 1
**Verbascoside**	1.9 ± 0.1	4.2 ± 0.1	2.48 ± 0.04	2.09 ± 0.08	5.7 ± 0.2

[a] TEAC values expressed in relation to gallic acid**.**
[b] TEAC values expressed in relation to Trolox.

Table S4. Content of polyphenols (mg) per g of olive leaves determined in liquid extracts before and upon lyophilization.

	Sample 5		Sample 7	
	**Liquid extract**	**Lyophilized powder**	**Liquid extract**	**Lyophilized powder**
**Hydroxytyrosol**	7.4 ± 0.7	2.9 ± 0.1	6.4 ± 0.3	1.6 ± 0.1
**Oleuropein**	485 ± 56	167 ± 1	202 ± 6	40 ± 1
**Pinoresinol**	3.6 ± 0.3	1.45 ± 0.01	2.3 ± 0.2	0.58 ± 0.01
**Verbascoside**	11.6 ± 0.3	4.35 ± 0.02	7.5 ± 0.5	1.88 ± 0.01
**Rutin**	5.0 ± 0.5	3.0 ± 0.1	4.4 ± 0.4	2.5 ± 0.1
**Quercetin**	1.4 ± 0.1	0.70 ± 0.01	1.2 ± 0.1	0.59 ± 0.1
**Luteolin**	1.7 ± 0.1	0.83 ± 0.03	4.9 ± 0.2	1.45 ± 0.04

a)

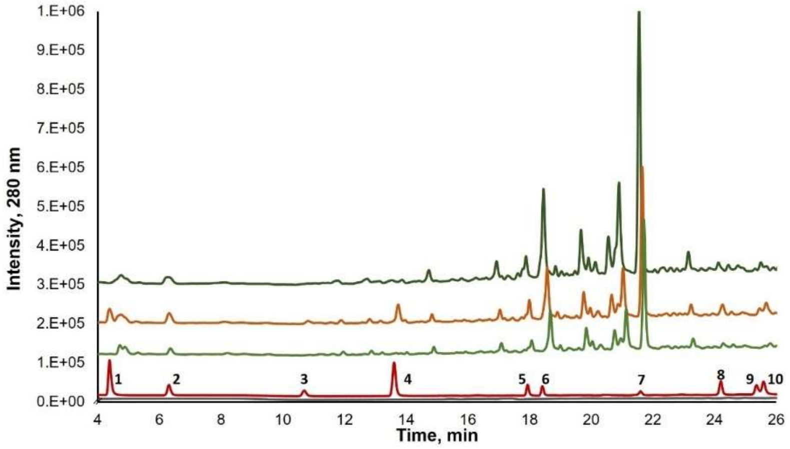

b)

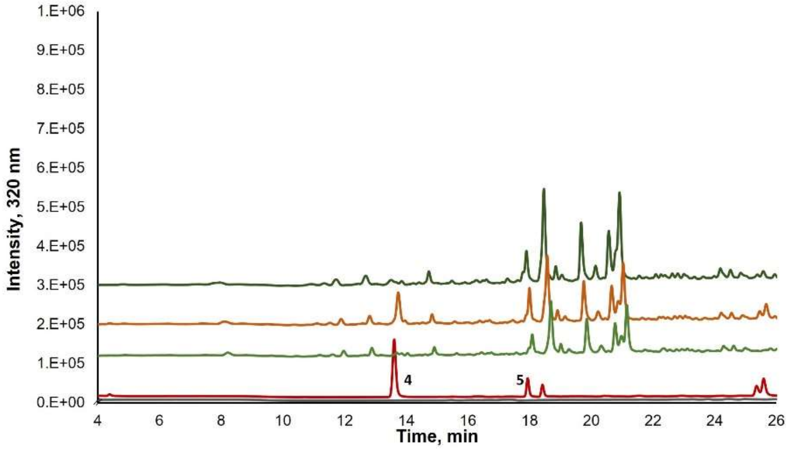

c)

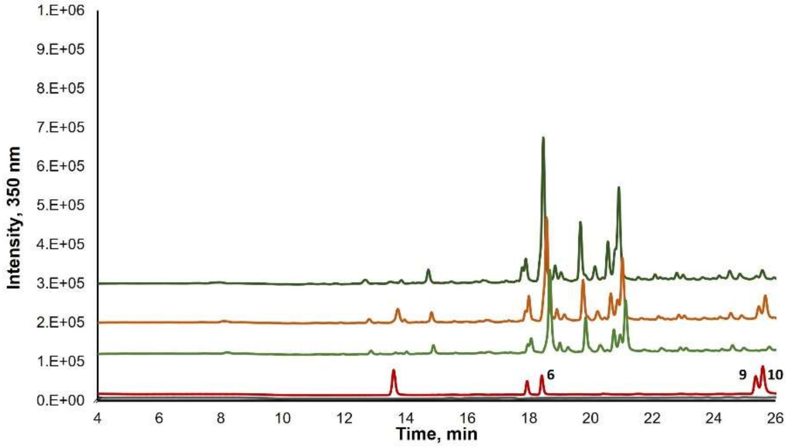

Figure S1. Chromatograms at (a) 280 nm, (b) 320 nm and (c) 350 nm depicting: a standard solution containing 10 mg L^{-1} of the following compounds (red line): 1) gallic acid, 2) hydroxytyrosol, 3) catechin, 4) caffeic acid, 5) verbascoside, 6) rutin, 7) oleuropein, 8) pinoresinol, 9) quercetin, and 10) luteolin; 50% (v/v) EtOH extract from sample 5, undiluted (dark green line) and diluted 2× (light green line) and, for the same extract, diluted 2× supplemented with 5 mg L^{-1} of each compound (orange line). Compounds 1-3, 7, 8 were monitored at 280 nm; compounds 4 and 5 were monitored at 320 nm; compounds 6, 9, 10 were monitored at 350 nm. The grey line represents the injection of mobile phase.

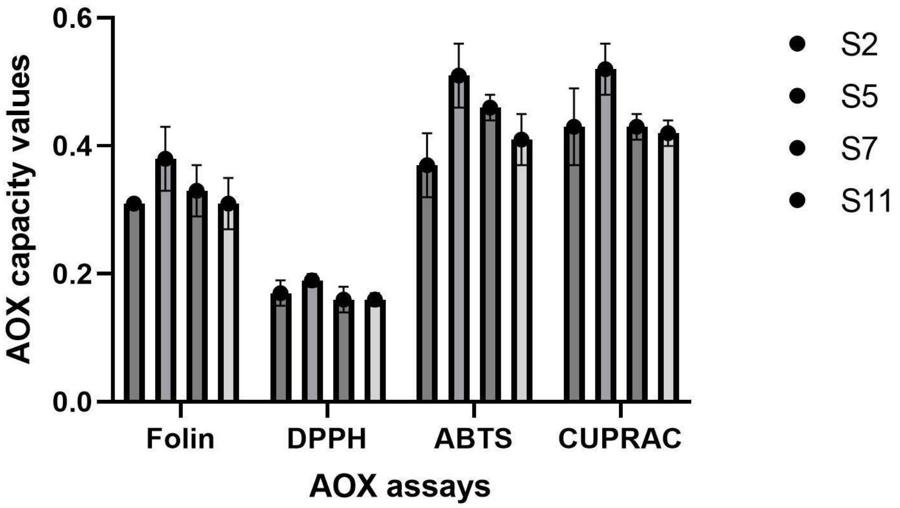

Figure S2. Antioxidant (AOX) capacity values (mean ± SD) for samples 2, 5, 7 and 11 as determined by Folin, DPPH, ABTS and CUPRAC methods.
